# Exploring the association between BMI and mortality in Australian women and men with and without diabetes: the AusDiab study

**DOI:** 10.1007/s00125-019-4830-4

**Published:** 2019-02-26

**Authors:** Syeda F. Zahir, Alison Griffin, J. Lennert Veerman, Dianna J. Magliano, Jonathan E. Shaw, Kim-Anh Lê Cao, Ahmed M. Mehdi

**Affiliations:** 1 The University of Queensland Diamantina Institute, Faculty of Medicine, The University of Queensland, Level 6, Translational Research Institute, 37 Kent Street, Woolloongabba, QLD 4102 Australia; 2 Independent Researcher, Brisbane, QLD Australia; 3 School of Medicine, Griffith University, Gold Coast, QLD Australia; 4 Baker Heart and Diabetes Institute, Melbourne, VIC Australia; 5 Melbourne Integrative Genomics, School of Mathematics and Statistics, University of Melbourne, Melbourne, VIC Australia

**Keywords:** Collider bias, Diabetes, Men, Mortality, Obesity paradox, Women

## Abstract

**Aims/hypothesis:**

There is conflicting evidence about the obesity paradox—the counterintuitive survival advantage of obesity among certain subpopulations of individuals with chronic conditions. It is believed that results supporting the obesity paradox are due to methodological flaws, such as collider bias. The aim of this study was to examine the association between obesity and mortality in Australian men and women. In addition, we explored whether obesity would appear to be protective if the analysis was restricted to a subpopulation with disease, and to discuss the potential role of collider bias in producing such a result.

**Methods:**

The examined cohort included 10,575 Australian adults (4844 men and 5731 women) aged 25–91 years who were recruited for the AusDiab baseline survey in 1999 and followed-up through 2014. The main predictor variable was BMI categorised as normal weight (18.5 to <25 kg/m^2^), overweight (25 to <30 kg/m^2^) and obese (≥30 kg/m^2^), and the outcome of interest was all-cause mortality. Hazard ratios were estimated from Cox proportional hazards regression models in the entire cohort and then in subpopulations with and without diabetes.

**Results:**

A total of 1477 deaths occurred during 145,384 person-years (median 14.6 years) of follow-up. Mortality was higher in obese than in normal-weight individuals for the full population (HR 1.18; 95% CI 1.05, 1.32). When an interaction between diabetes status and BMI category was added to the model, there was no evidence of an interaction between BMI and diabetes status (*p =* 0.92). When participants with and without diabetes were analysed separately, there was no evidence of an association between obesity and mortality in those with diabetes (HR 0.91; 95% CI 0.62, 1.33).

**Conclusions/interpretation:**

In the entire AusDiab cohort, we found a significantly higher mortality among obese participants as compared with their normal-weight counterparts. We found no difference in the obesity–mortality association between individuals with and without diabetes.

**Electronic supplementary material:**

The online version of this article (10.1007/s00125-019-4830-4) contains peer-reviewed but unedited supplementary material, which is available to authorised users.

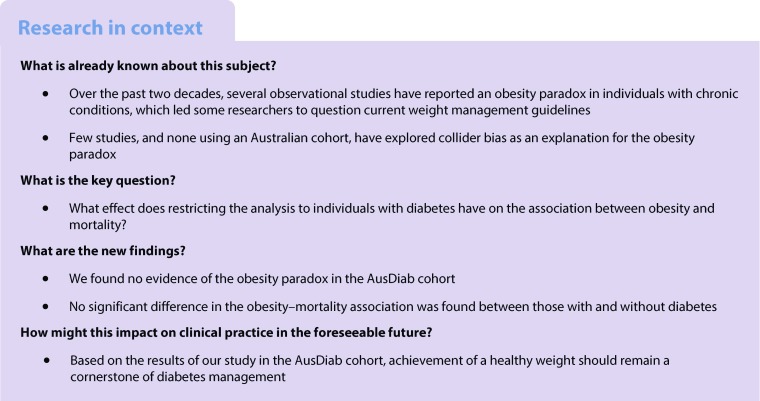



## Introduction

There is conflicting evidence on the ‘obesity paradox’, the putative survival advantage of obesity among individuals with chronic conditions [1, 2]. Current weight management guidelines recommend that a BMI <25 kg/m^2^ should be maintained [3]. However, in light of these paradoxical findings, some researchers have advocated the need for revision of these guidelines for individuals with chronic diseases [4]. It is therefore crucial to understand whether these findings reflect true causal effects or result from methodological flaws in the studies reporting the paradox.

Although many hypotheses have been put forward to explain the counterintuitive survival advantage of obesity, the evidence is still inconclusive [5–7]. Collider bias has emerged as the most recent explanation of the obesity paradox [2, 8]. Collider bias is defined as bias due to conditioning on a variable affected by exposure and sharing common causes with the outcome. While previous studies have shown that bias caused by stratification by diabetes status might be responsible for the obesity paradox in people with chronic conditions [2, 8], no such study has been conducted in an Australian cohort. Our primary research objective was to examine the association between obesity and mortality in Australian men and women and to explore the effect of restricting the analysis to individuals with diabetes. In addition, we explored the BMI–mortality association in individuals with and without diabetes relative to normal-weight individuals without diabetes.

## Methods

### Study participants

The cohort included 10,575 adult participants (4844 men and 5731 women, aged 25 to 91 years) enrolled in the Australian Diabetes, Obesity and Lifestyle (AusDiab) study. A flow chart of study population derivation is given in ESM Fig. 1. Participants were followed from the date of their baseline examination until 31 December 2014, or until death if sooner. All participants provided written informed consent and the study protocol was approved by the ethics committee of the Baker Heart and Diabetes Institute. Additional details are presented in the ESM Methods.

### Variables

BMI at baseline was the primary exposure variable for this study. During biomedical examination, standard anthropometric measures were obtained by trained staff. Participants were classified as per WHO guidelines as normal weight (18.5 to <25 kg/m^2^), overweight (25 to <30 kg/m^2^) and obese (≥30 kg/m^2^). Participants were classified as having diabetes based on venous plasma glucose levels as recommended by WHO (ESM Table 1) [9], or if they were currently being treated with insulin or oral glucose-lowering drugs (see ESM variables in ESM Methods for details). The outcome of this study was all-cause mortality, which was defined as death from any cause until 31 December 2014. Mortality status was identified by linking the AusDiab data to the Australian National Death Index.

### Statistical analysis

Cox proportional hazards regression was used to model the association between BMI category and all-cause mortality. Models were adjusted for sex, level of education, weekly income, smoking status, physical activity and cluster of census collection district area. The baseline hazard function in the model was stratified by age and marital status, as the proportional hazards assumption was satisfied after their inclusion.

We estimated hazard ratios for (1) the full population; (2) for participants with and without diabetes separately; (3) for men and women separately; and (4) to compare mortality for each BMI/diabetes status relative to ‘normal-weight participants without diabetes’, the addition of diabetes status to the model with an interaction term with BMI category. Evidence for effect modification of the association between BMI category and mortality by diabetes status was examined by testing the significance of this interaction term (please see ESM statistical analysis for additional details).

Sensitivity analyses included consideration of diabetes status, BMI, smoking and physical activity as time-dependent variables. Variables used in time-varying analyses were measured at baseline, and then at the two follow-up surveys (2004–2005 and 2011–2012). Additional sensitivity analyses of our final models (1) and (2) were conducted after exclusion of ever smokers; exclusion of deaths in the first 3 and 5 years of follow-up; exclusion or reclassification of individuals with impaired fasting glucose (IFG) and impaired glucose tolerance (IGT) as having diabetes. All data analyses were conducted using Stata (version 13.1, StataCorp, College Station, TX, USA) or R (version 3.4.0, https://cran.r-project.org/src/base/R-3/R-3.4.0.tar.gz).

## Results

In total, 10,575 participants (54.2% women) were included in the analysis, of whom 860 (8.1%) had diabetes at baseline (ESM Tables 2, 3).

### Association between BMI and all-cause mortality

Participants were observed for a total of 145,384 person-years (median 14.6 years). A total of 1477 deaths occurred during follow-up, giving a mortality rate of 102 per 10,000 person-years. The number of deaths by diabetes status and BMI category are reported in ESM Table 4.

For the full population, mortality was higher in obese than in normal-weight individuals (HR 1.18; 95% CI 1.05, 1.32) (Table 1). When diabetes status and an interaction term between diabetes and BMI category were included in the model, there was no evidence of an interaction between BMI and diabetes status (*p* = 0.92) (ESM Table 5). When participants with and without diabetes were analysed separately, obesity was found to be associated with higher mortality in those without diabetes (HR 1.16; 95% CI 1.01, 1.34). However, there was no evidence of an obesity–mortality association in those with diabetes (HR 0.91; 95% CI 0.62, 1.33) (Table 1).

**Table 1:** Hazard ratios for all-cause mortality by BMI category for all participants and participants without and with diabetes

Population	Deaths/*n*	BMI category: HR (95% CI)			*p* value
		Normal weight	Overweight	Obese	
Total	1438/10394	1.00	0.97 (0.87,1.09)	1.18 (1.05,1.32)	0.001
Without diabetes	1131/955*7*	1.00	1.00 (0.88,1.13)	1.16 (1.01,1.34)	0.06
With diabetes	307/837	1.00	0.86 (0.60,1.21)	0.91 (0.62,1.33)	0.65

Adjusted for sex, educational attainment, weekly income, smoking status, physical activity, cluster, and strata of age group and marital status

Similar results were obtained in sensitivity analyses when (1) time-varying covariates were included in the model; (2) analysis was restricted to never smokers; (3) after excluding deaths within the first 3 and then 5 years; (4) after excluding or reclassifying individuals with IFG and IGT as having diabetes; and when BMI was used as a continuous variable with relaxation of linearity (ESM Tables 6–10, ESM Fig. 2).

### Sex-specific analysis

For the full population (participants with and without diabetes), obesity appeared to be associated with higher mortality in women (HR 1.31; 95% CI 1.07, 1.61), but not in men (HR 1.10; 95% CI 0.91, 1.34) (Table 2). However, the interaction term was non-significant (*p* = 0.38), indicating no statistical evidence of a difference between men and women with regard to the association between obesity and mortality.

**Table 2:** Sex-specific hazard ratios for all-cause mortality by BMI category in all participants and participants without and with diabetes

Population	Women				Men			
	BMI category: HR (95% CI)			*p* value	BMI category: HR (95% CI)			*p* value
	Normal weight	Overweight	Obese		Normal weight	Overweight	Obese	
Total	1.00	0.96 (0.81,1.15)	1.31 (1.07,1.61)	0.002	1.00	1.01 (0.85,1.20)	1.10 (0.91,1.34)	0.57
Without diabetes	1.00	0.98 (0.80,1.21)	1.32 (1.05,1.64)	0.01	1.00	1.05 (0.86,1.28)	1.08 (0.84,1.38)	0.82
With diabetes	1.00	0.79 (0.47,1.34)	0.93 (0.54,1.62)	0.56	1.00	0.80 (0.48,1.35)	0.74 (0.43,1.28)	0.55

Adjusted for sex, educational attainment, weekly income, smoking status, physical activity, cluster, and strata of age group and marital status

## Discussion

Our findings illustrate that obesity was associated with higher mortality in the entire cohort, and there was no evidence of a protective effect of obesity on mortality when the analysis was restricted to individuals with diabetes. There was no statistical evidence that the obesity–mortality association was different between those with and without diabetes (*p* = 0.92 for interaction between BMI and diabetes status). However, if the study had only included participants with diabetes, these findings could have been interpreted as evidence of a lack of association between obesity and mortality in individuals with diabetes.

Studies reporting the obesity paradox have been criticised for restricting analysis to individuals with disease [2, 8], which could result in a form of selection bias termed collider bias. When analyses are conducted on a selected group of individuals (including people with diabetes and ignoring those without diabetes in the same population), conditioning on the collider (diabetes in this case) occurs, which affects the exposure–outcome association in an unpredictable way. Our study is consistent with previous studies that confirm that restricting analysis in this way could lead to an apparent protective association or loss of association between obesity and mortality. However, due to the small sample size and non-significant interaction term, we are unable to conclude that the lack of association is due to collider bias or that obesity is behaving differently in those with or without diabetes. There is some scepticism in the field about collider bias, as the factor biasing the result is unknown and hence cannot be accounted for [10]. Previous studies have demonstrated that the obesity paradox can be only partially explained by collider bias [6, 11].

While in this study it appeared that there was no association between obesity and mortality among men, the non-significant interaction term for sex indicated that the significant association between obesity and mortality in the whole population likely applied equally to men and women. The differences in the obesity–mortality association between the sexes in this study might have been due to chance. A meta-analysis previously confirmed an association between obesity and higher mortality in both men and women [12].

Strengths of our study include measured weight and height, long-term follow-up and detailed assessment of clinical and socioeconomic factors. Limitations of the study include the relatively small number of deaths, the use of BMI as a surrogate measure of adiposity, self-reported smoking status and the inclusion of smokers in analyses. In addition, lack of data at baseline precluded adjustment for current medical history or exclusion of participants with chronic conditions, which could result in some bias due to reverse causation.

To summarise, studies reporting the obesity paradox present a confusing message for clinicians and policy makers, leading to a risk of misinforming obese individuals about healthy lifestyle management plans. In this study we found no evidence of the obesity paradox in individuals with diabetes in the AusDiab cohort, and found no difference between participants with and without diabetes with regard to the association between obesity and mortality.

## Electronic supplementary material

ESM (PDF 359 kb)

## Data Availability

The data that support the findings of this study are available from AusDiab but restrictions apply to the availability of these data, which were used under license for the current study, and so are not publicly available.

## Abbreviations

AusDiab: Australian Diabetes, Obesity and Lifestyle Study
IFG: Impaired fasting glucose
IGT: Impaired glucose tolerance
