# Supplementary material for: Exploring the association between BMI and mortality in Australian women and men with and without diabetes: the AusDiab study
Source: Diabetologia. 2019 Feb 26;62(5):754–8. doi: 10.1007/s00125-019-4830-4 (PMC6450848; doi:10.1007/s00125-019-4830-4)
Supplement: Supplementary file 1 — (PDF 359 kb) [file 125_2019_4830_MOESM1_ESM.pdf]

Electronic supplementary material

ESM Methods

ESM study participants

The Australian Diabetes, Obesity and Lifestyle (AusDiab) study is a population based longitudinal study, with a nationwide representative sample of adults 25 years and older. This study had three phases, with a baseline survey conducted in 1999-2000 using a nationwide cluster sampling method, from 42 randomly selected districts involving six states and the Northern Territory. The data collection method consisted of a household questionnaire, interviews and a biomedical examination. Of 20,347 eligible participants, 11,247 participants (55.3%) completed the household questionnaire and attended the biomedical examination and formed the sample for the baseline study. Participants of the baseline study were invited for second and third waves of the survey undertaken in 2004-2005 and 2011-2012, respectively. Further details of the AusDiab study design and methods have been published elsewhere [1, 2]. The data used in the present study were retrieved from the AusDiab baseline survey.

ESM variables

BMI category at baseline was the primary exposure variable for this study. Height was measured to the nearest 0.5 cm using a stadiometer and weight was measured to the nearest 0.5 kg using a mechanical beam balance scale. BMI was calculated as weight in kilograms divided by square of height in meters (BMI=kg/m^2). Diabetes was defined by the WHO classification of diabetes mellitus [3], based on the 75 gm 2-hour oral glucose tolerance test (Table 1), venous plasma glucose levels (fasting plasma glucose level ≥7.0 or 2-hour glucose level ≥11.1 mmol/l). Additionally, participants currently treated with insulin or oral hypoglycaemic agents were classified as having diabetes. The primary outcome of this study

was all-cause mortality which was identified by linking the AusDiab data to the Australian National Death Index (NDI). The accuracy of the NDI in identifying the vital status of the Australian population has been reported previously [4]. Demographic and other variables included age, sex, marital status, education level, weekly income, smoking and physical activity status. Information about demographic characteristics was collected through interviewer led household and general questionnaires. Age was categorized into: 25-34, 35-44, 45-54, 55-59, 60-64, 65-69, 70-74, 75-79, 80-84, and ≥85 year age groups. Smoking status was assessed based on self-reported responses obtained from the general questionnaire at baseline and categorized as current smoker, ex-smoker or never smoker. Self-reported physical activity classification was based on proposed national standards for the measurement of physical activity (PA) developed by the Australian Institute of Health and Welfare (AIHW) and categorized as sedentary (0 min PA time/week), insufficient (1 -149min PA time/week) or sufficient (over 149 min PA time/week)[5]. The level of educational attainment was categorized as never attended school/primary school, some high school, completed high school, or university/TAFE. Marital status was categorized as married/de facto; separated/divorced/widowed; or never married. Weekly income was categorized as ≥$1500, $800-1499, $400-799, or $0-399.

ESM statistical analysis

Individuals still alive at the end of follow-up were censored and tied survival times were broken using Breslow's method. All variables were treated as categorical. Interaction terms between BMI and each of age, smoking status, and physical activity were examined, none were significant at $p<0.05$ and hence these interactions were not included in the final model. The proportional hazards (PH) assumption was tested by examining the relationship between the scaled Schoenfeld residuals and survival time and the overall fit of the models was assessed by examining the Cox-Snell residuals. Complete case analysis was used initially, and

analyses were then repeated using multiple imputation of missing values. No substantial differences were noted and HR and 95% CI using complete case analysis are presented.

Missing values and multiple imputation:

As noted in the flow diagram of study population derivation (ESM Figure 1), participants with missing values for BMI, diabetes status, smoking status and date of death were excluded from the study, leaving 10,575 participants (with complete values for these variables) available for analysis. Of these, 10,394 participants had no missing values and were included in complete case analysis, while 181 had missing values for weekly income (n=156), physical activity (n=22), marital status (n=4) or educational attainment (n=3), some participants had missing values for more than one variable. Multinomial logistic regression on the remaining predictor variables, the Nelson-Aalen estimator of the cumulative hazard function and the event indicator was used to impute missing values [6]. Final models were fitted separately on each of 30 imputed datasets and results were combined according to the combination rules by Rubin [7].

ESM Tables

ESM Table 1. WHO classification for diagnostic criteria of diabetes and intermediate hyperglycaemia based on plasma glucose levels

Status	Fasting plasma glucose level (mmol/l)	And/or	2-hour plasma glucose level (mmol/l)
Normal glucose tolerance (NG)	<6.1	and	<7.8
Impaired fasting glucose (IFG)	6.1-6.9	and	<7.8
Impaired glucose tolerance (IGT)	<7.0	and	7.8-11.0
Diabetes	≥7.0	or	≥11.1

Mmol/l: Millimoles per litre   Cited and modified from [3]

Descriptive tables

ESM Table 2. Baseline characteristics of participants of the AusDiab cohort by BMI category and diabetes status

	**Participants without diabetes**				**Participants with diabetes [a]**			
	**Normal weight**	**Overweight**	**Obese**	**All**	**Normal weight**	**Overweight**	**Obese**	**All**
***n* (male %)**	3736 (36.2)	3994 (55.5)	1985 (41.2)	9715 (45.1)	150 (50.0)	317 (59.0)	393(49.4)	860 (53.0)
**Age, years: mean (SD)**	48.7(14.4)	52.4 (14.1)	51.1 (12.8)	50.7 (14.0)	68.5 (12.5)	64.0 (11.5)	60.6 (11.8)	63.2 (12.2)
**Marital status %**								
Married/De facto	75.8	78.9	77.3	77.4	60.0	69.0	69.5	67.6
Separated/Divorced/Widowed	14.0	14.1	15.7	14.4	34.7	26.9	21.9	26.0
Never married	10.3	7.0	7.1	8.3	5.3	4.1	8.7	6.4
**Education attainment %**								
Never attended school/Primary	4.5	6.3	6.9	5.7	18.7	17.4	16.5	17.2
Some high school	33.5	36.8	45.5	37.3	43.3	41.8	45.6	43.8
Completed high school	20.4	19.0	17.3	19.2	15.3	14.2	15.3	14.9
University/ TAFE	41.6	37.9	30.3	37.8	22.7	26.6	22.7	24.1
**Weekly income %**								
$ 1500+	17.5	18.2	15.3	17.3	7.7	6.8	7.8	7.4
$ 800-1499	29.6	28.6	27.6	28.8	10.5	15.4	17.7	15.6
$400-799	29.7	28.0	30.4	29.1	31.5	32.8	27.8	30.3
$0-399	23.2	25.3	26.8	24.8	50.4	45.0	46.8	46.7
**BMI kg/m^2: mean (SD)**	22.6 (1.6)	27.2 (1.4)	33.9 (3.8)	26.8 (4.7)	22.9 (1.6)	27.6 (1.4)	35.2(4.8)	30.3 (5.9)
**Waist circumference, cm, mean (SD)**								
**Men**	86.9 (6.4)	97.6 (6.4)	111.4 (8.9)	96.9 (10.9)	89.7 (5.7)	101.2 (6.4)	116.3 (9.9)	105.7 (12.7)
**Women**	75.2 (6.5)	86.8 (6.8)	101.5 (10.0)	84.8 (12.6)	80.1 (7.2)	92.1 (6.7)	108.5 (11.5)	97.9 (14.6)
**Physical activity in past week (PA) % [b]**								
Sedentary (0 min PA)	14.1	16.0	21.9	16.5	15.3	23.1	30.3	25.0
Insufficient (b/w 0 and 149 min PA)	31.2	28.6	32.8	30.5	40.0	30.4	29.5	31.7
Sufficient (>149 min PA)	54.7	55.4	45.3	53.1	44.7	46.5	40.2	43.3
**Smoking %**								
Current-smoker	17.3	14.8	15.1	15.8	12.7	13.6	12.0	12.7
Ex-smoker	24.4	31.0	30.7	28.4	38.0	37.2	39.2	38.3
Non-smoker	58.3	54.2	54.2	55.8	49.3	49.2	48.9	49.1
**Systolic BP, mmHg: mean (SD)**	123.2 (17.8)	130.8 (17.8)	133.4 (16.8)	128.4 (18.1)	142.7 (22.8)	144.3 (19.7)	145.2 (18.6)	144.4 (19.8)
**Hypertension %[c]**	19.1	33.5	41.8	29.7	60.7	73.7	72.1	70.7

[a] Based on World Health Organization criteria [3], [b] based on Australian Institute of Health and Welfare criteria[5], [c] based on ≥140/90 mmHg and/or taking blood pressure-lowering medication

ESM Table 3. Age at baseline of the 10, 575 participants of the AusDiab study, by BMI category and diabetes status

Diabetes status	BMI category	*n*	Mean	SD	Range
Without diabetes	Normal weight	3736	48.7	14.4	(25, 89)
	Overweight	3994	52.4	14.1	(25, 91)
	Obese	1985	51.1	12.8	(25, 90)
With diabetes	Normal weight	150	68.5	12.5	(29, 89)
	Overweight	317	64.1	11.5	(35, 91)
	Obese	393	60.6	11.8	(25, 87)

ESM Table 4. Sample size (*n*) and number of events (deaths) by BMI category and diabetes status for 10,394 participants of the AusDiab study examined in the multivariable model

		**BMI Category**							
		**Normal weight**		**Overweight**		**Obese**		**Total**	
		**Events**	***n***	**Events**	***n***	**Events**	***n***	**Events**	***n***
**Total**	**Persons**	443	3820	645	4242	350	2332	1438	10394
	Men	218	1412	396	2380	155	998	769	4790
	Women	225	2408	249	1862	195	1334	669	5604
Without diabetes	Persons	370	3677	537	3933	224	1947	1131	9557
	Men	180	1340	328	2198	90	807	598	4345
	Women	190	2337	209	1735	134	1140	533	5212
With diabetes	Persons	73	143	108	309	126	385	307	837
	Men	38	72	68	182	65	191	171	445
	Women	35	71	40	127	61	194	136	392

Mortality relative to 'normal weight without diabetes'

ESM Table 5. Hazard ratios and 95% confidence intervals for all-cause mortality by weight status and diabetes status relative to normal weight without diabetes

Weight and diabetes status	HR	95% CI	*p*-value
Normal weight without diabetes	1.00	(Reference)	
Overweight without diabetes	0.98	0.87,1.11	0.80
Obese without diabetes	1.12	0.98,1.29	0.10
Normal weight with diabetes	1.43	1.09,1.89	0.01
Overweight with diabetes	1.31	1.05,1.63	0.02
Obese with diabetes	1.50	1.20,1.87	<0.001

Adjusted for sex, educational attainment, weekly income, smoking status, physical activity, cluster, and strata of age group and marital status.
The *p*-value for the interaction term between BMI and diabetes status is 0.92

Sensitivity Analyses

ESM Table 6. Hazard ratios and 95% confidence intervals for all-cause mortality by BMI category for all participants and participants with diabetes considering diabetes status, BMI category, smoking status and physical activity as time-varying covariates

Population	Time varying covariates	BMI Category: HR (95% CI)			*p*-value
		Normal weight	Overweight	Obese	
Total	Diabetes	1.00	0.99 (0.87,1.12)	1.15 (0.99,1.33)	0.02
	Diabetes, BMI	1.00	0.94 (0.83,1.06)	1.03 (0.89,1.19)	0.19
	Diabetes, smoking, PA	1.00	0.97 (0.86,1.10)	1.11 (0.96,1.29)	0.02
	Diabetes, smoking, BMI, PA	1.00	0.95(0.84,1.07)	1.03 (0.89,1.19)	0.19
With diabetes	Diabetes	1.00	0.91 (0.67,1.22)	1.00 (0.74,1.35)	0.48
	Diabetes, BMI	1.00	0.82(0.61,1.10)	0.86 (0.64,1.16)	0.65
	Diabetes, smoking, PA	1.00	0.86 (0.64,1.16)	0.90 (0.67,1.23)	0.48
	Diabetes, BMI, smoking, PA	1.00	0.83 (0.62,1.11)	0.85 (0.63,1.14)	0.65

Adjusted for sex, educational attainment, weekly income, smoking status, physical activity, strata of age group and marital status.

ESM Table 7. Hazard ratios and 95% confidence intervals for all-cause mortality in participants with diabetes excluding deaths in the first 3 and then 5 years.

Population	Excluding	Deaths/*n*	BMI Category: HR (95% CI)			*p*-value
			Normal	Overweight	Obese	
With diabetes		307/837	1.00	0.86 (0.60,1.21)	0.91 (0.62,1.33)	0.65
	First 3yrs deaths	252/782	1.00	0.89 (0.58,1.37)	0.87 (0.59,1.29)	0.80
	First 5yrs deaths	216/746	1.00	0.77 (0.48,1.23)	0.85 (0.57,1.28)	0.54

Adjusted for sex, educational attainment, weekly income, smoking status, physical activity, cluster, and strata of age group and marital status.

ESM Table8. Hazard ratios and 95% confidence intervals for all-cause mortality by BMI category and diabetes status while restricting analysis to never smokers

Population	Deaths/*n*	BMI Category: HR (95% CI)			*p*-value
		Normal weight	Overweight	Obese	
Total	698/5742	1.00	0.97 (0.81,1.16)	1.17 (0.96,1.42)	0.07
Without diabetes	563/5332	1.00	0.98 (0.80,1.20)	1.13 (0.87,1.46)	0.40
With diabetes	135/410	1.00	0.79 (0.45,1.39)	0.91 (0.53,1.56)	0.64

Adjusted for sex, educational attainment, weekly income, smoking status, physical activity, cluster, and strata of age group and marital status.

ESM Table 9. Hazard ratio and 95% confidence intervals for all-cause mortality by BMI category and diabetes status after reclassifying individuals with impaired fasting glucose and impaired glucose tolerance as having diabetes

	Deaths/*n*	BMI Category: HR (95% CI)			*p*-value
		Normal weight	Overweight	Obese	
Total	1438/10394	1.00	0.97 (0.87,1.09)	1.18 (1.05,1.32)	0.001
Without diabetes	728/7663	1.00	1.03 (0.87,1.20)	1.23 (1.00,1.51)	0.11
With diabetes	710/2731	1.00	0.88 (0.72,1.06)	0.97 (0.79,1.19)	0.27

Adjusted for sex, educational attainment, weekly income, smoking status, physical activity, cluster, and strata of age group and marital status.

ESM Table10. Hazard ratio and 95% confidence intervals for all-cause mortality by BMI category and diabetes status after excluding individuals with impaired fasting glucose and impaired glucose tolerance

Population	Deaths/*n*	BMI Category: HR (95% CI)			*p*-value
		Normal weight	Overweight	Obese	
Total	1035/8500	1.00	0.99 (0.87,1.12)	1.26 (1.07,1.47)	0.002
Without diabetes	728/7663	1.00	1.03 (0.87,1.20)	1.23 (1.00,1.51)	0.11
With diabetes	307/837	1.00	0.86 (0.60,1.21)	0.91 (0.62,1.33)	0.65

Adjusted for sex, educational attainment, weekly income, smoking status, physical activity, cluster, and strata of age group and marital status.

ESM Figures

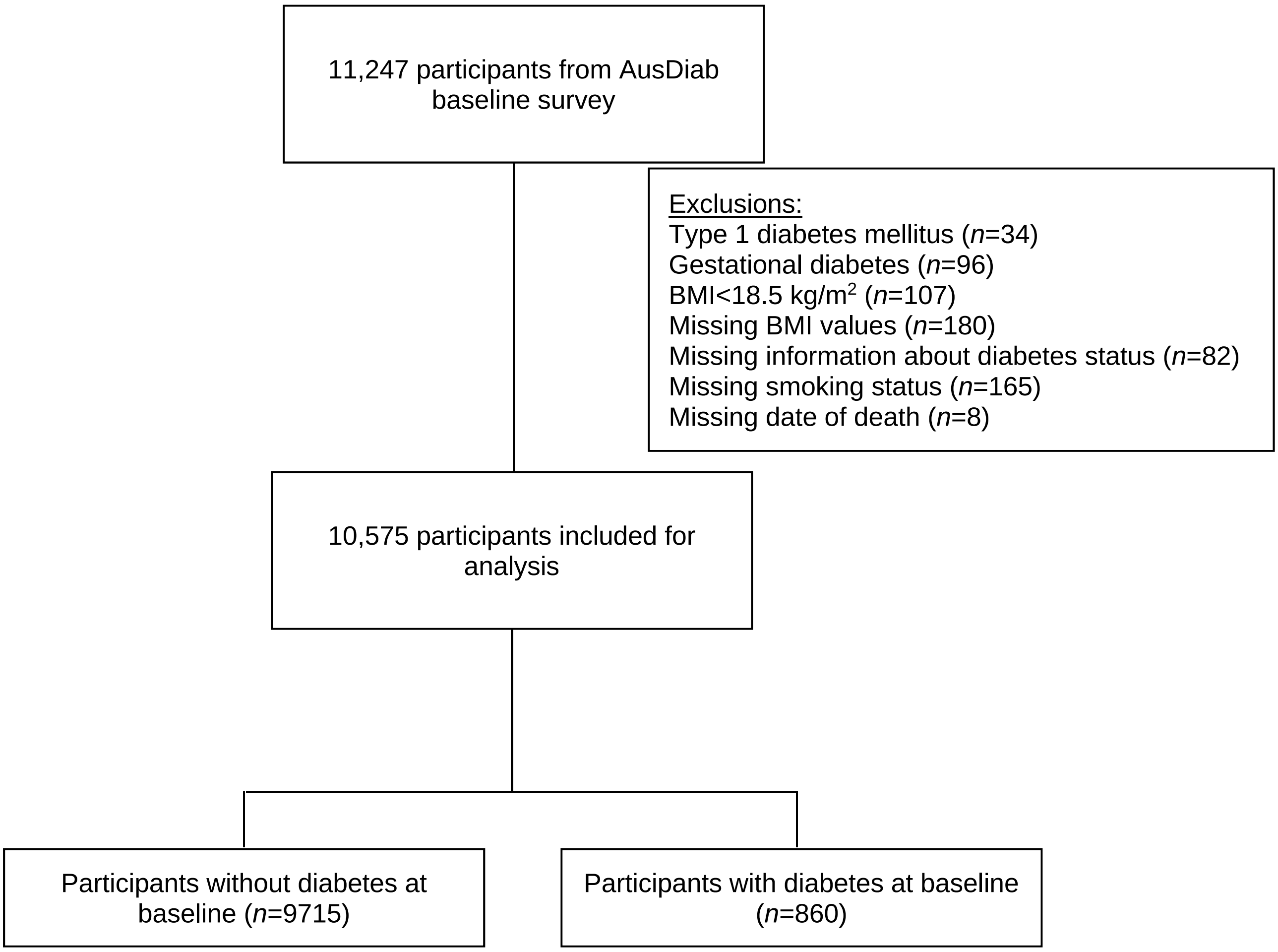

ESM Figure 1: Flow diagram of study population derivation

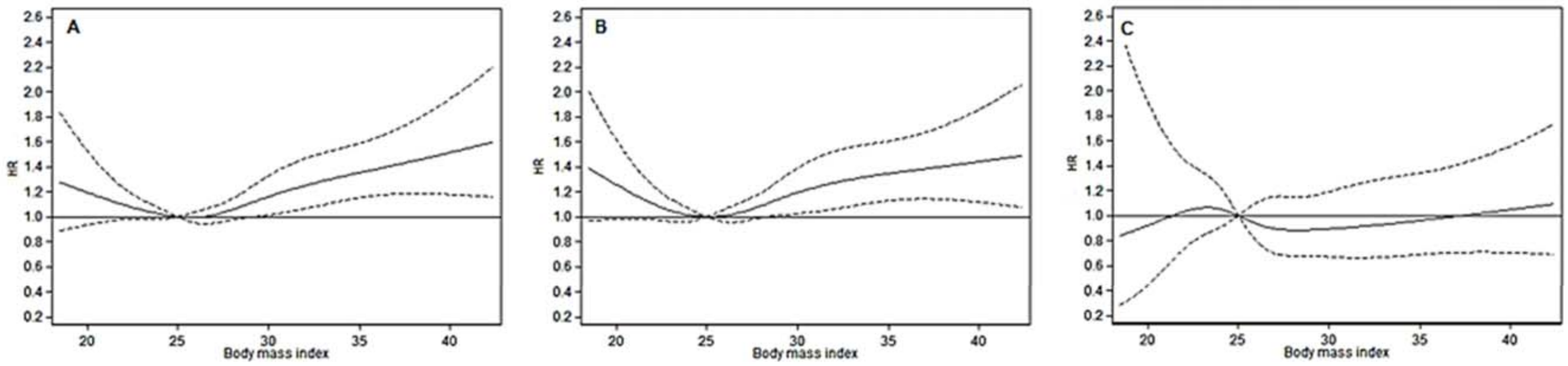

ESM Figure 2: Plots displaying the association between BMI and mortality by fitting restricted cubic spline model for (A) entire cohort; (B) individuals without diabetes; and (C) individuals with diabetes. Non-linear relationship between BMI and mortality is displayed with solid black line. Dashed lines represent 95% CI

References:

[1] Dunstan DW, Zimmet PZ, Welborn TA, et al. (2002) The Australian Diabetes, Obesity and Lifestyle Study (AusDiab)--methods and response rates. Diabetes Res Clin Pract 57: 119-129

[2] Magliano DJ, Barr EL, Zimmet PZ, et al. (2008) Glucose indices, health behaviors, and incidence of diabetes in Australia: the Australian Diabetes, Obesity and Lifestyle Study. Diabetes Care 31: 267-272

[3] World Health Organization (1999) Definition, diagnosis and classification of diabetes mellitus and its complications. Part 1: diagnosis and classification of diabetes mellitus. In. Department of Noncommunicable Disease Surveillance, WHO, Geneva

[4] Magliano D, Liew D, Pater H, et al. (2003) Accuracy of the Australian National Death Index: comparison with adjudicated fatal outcomes among Australian participants in the Long-term Intervention with Pravastatin in Ischaemic Disease (LIPID) study. Aust N Z J Public Health 27: 649-653

[5] Armstrong T, Bauman AE, Davies J (2000) Physical activity patterns of Australian adults: results of the 1999 National Physical Activity Survey. Australian Institute of Health and Welfare

[6] White IR, Royston P (2009) Imputing missing covariate values for the Cox model. Stat Med 28: 1982-1998

[7] Rubin DB (2004) Multiple imputation for nonresponse in surveys. John Wiley & Sons
